# Uptake of influenza vaccination in pregnancy amongst Australian Aboriginal and Torres Strait Islander women: a mixed-methods pilot study

**DOI:** 10.1186/s13104-015-1147-3

**Published:** 2015-04-29

**Authors:** Kerry-Ann F O’Grady, Melissa Dunbar, Linda G Medlin, Kerry K Hall, Maree Toombs, Judith Meiklejohn, Lisa McHugh, Peter D Massey, Amy Creighton, Ross M Andrews

**Affiliations:** Queensland Children’s Medical Research Institute, Queensland University of Technology, Level 7 Centre for Child Health Research, South Brisbane, Queensland Australia; University of Queensland Rural Clinical School, School of Medicine, The University of Queensland, Toowoomba, Queensland Australia; Menzies School of Health Research, Charles Darwin University, Spring Hill, Queensland Australia; National Centre for Epidemiology and Population Health, Australian National University, Canberra, Australian Capital Territory Australia; Centre for Child Health Research, The University of Queensland, South Brisbane, Queensland Australia; Communicable Diseases Branch, Queensland Health, Herston, Queensland Australia; Hunter New England Population Health, New South Wales Health, Tamworth, New South Wales Australia

**Keywords:** Influenza, Vaccine, Pregnancy, Aboriginal and Torres Strait Islander Australian, Determinants, Uptake

## Abstract

**Background:**

Influenza infection during pregnancy causes significant morbidity and mortality. Immunisation against influenza is recommended during pregnancy in several countries however, there are limited data on vaccine uptake, and the determinants of vaccination, in pregnant Australian Aboriginal and/or Torres Islander women. This study aimed to collect pilot data on vaccine uptake and attitudes towards, and perceptions of, maternal influenza vaccination in this population in order to inform the development of larger studies.

**Methods:**

A mixed-methods study comprised of a cross-sectional survey and yarning circles (focus groups) amongst Aboriginal and Torres Strait Islander women attending two primary health care services. The women were between 28 weeks gestation and less than 16 weeks post-birth. These data were supplemented by data collected in an ongoing national Australian study of maternal influenza vaccination. Aboriginal research officers collected community data and data from the yarning circles which were based on a narrative enquiry framework. Descriptive statistics were used to analyse quantitative data and thematic analyses were applied to qualitative data.

**Results:**

Quantitative data were available for 53 women and seven of these women participated in the yarning circles. The proportion of women who reported receipt of an influenza vaccine during their pregnancy was 9/53. Less than half of the participants (21/53) reported they had been offered the vaccine in pregnancy. Forty-three percent reported they would get a vaccine if they became pregnant again. Qualitative data suggested perceived benefits to themselves and their infants were important factors in the decision to be vaccinated but there was insufficient information available to women to make that choice.

**Conclusions:**

The rates of influenza immunisation may continue to remain low for Aboriginal and/or Torres Strait Islander women during pregnancy. Access to services and recommendations by a health care worker may be factors in the lower rates. Our findings support the need for larger studies directed at monitoring and understanding the determinants of maternal influenza vaccine uptake during pregnancy in Australian Aboriginal and Torres Strait Islander women. This research will best be achieved using methods that account for the social and cultural contexts of Aboriginal and Torres Strait Islander communities in Australia.

## Background

Influenza infection during pregnancy is associated with increased morbidity, higher rates of hospitalization than the general population [1,2] and high mortality rates, particularly during pandemic periods [3]. Consequently, pregnant women are deemed a priority group for influenza vaccination both in Australia and overseas [4-6]. Furthermore maternal influenza vaccination during pregnancy appears to confer protection against influenza infection in young infants up to six months of age, a group for whom a licensed influenza vaccine is not available [7,8].

Aboriginal and/or Torres Strait Islander Australians are at increased risk of influenza and adverse influenza infection outcomes [9,10]. Given the increased risk, annual influenza vaccination is recommended in Australia, and the vaccine is free, for all Aboriginal and/or Torres Strait Island Peoples aged 15 years and older [11]. There are limited data available on uptake during pregnancy in this population. A large national study is underway in Australia (the FluMum Study) [12] however those data will be predominantly derived from women giving birth in major cities and may not reflect the experience of women in regional and rural areas.

In order to achieve high uptake an in-depth understanding of the determinants of vaccination is required through large studies that account for the heterogeneity of the Australian Aboriginal and Torres Strait Islander population [13,14]. To inform these studies, we conducted a mixed-methods pilot study of influenza vaccine uptake, and the factors influencing vaccine uptake, during pregnancy in Aboriginal and Torres Strait Islander women attending two Aboriginal and Torres Strait Islander specific primary health care centres in south-east Queensland, Australia.

## Methods

### Setting

The study was conducted from January to April 2014 in Caboolture and Toowoomba, Queensland. Both towns are within two hours drive of Brisbane, the capital city of Queensland, and are home to large Aboriginal and Torres Strait Islander communities. Both towns are serviced by Aboriginal and Torres Strait Islander specific primary health care services that include maternal and child health programs and have total clinic populations exceeding 3000 clients each.

### Design

The study utilised a mixed methods approach comprised of three components:

1. An analysis of data collected from women enrolled in the national FluMum study [12] at the Brisbane site.
2. A cross-sectional survey of women presenting to the two primary health care services.
3. Yarning circles (focus groups) [15] of women who had completed the cross-sectional survey outlined in step 2.

The study was approved by the Human Research Ethics Committee of the Queensland University of Technology (#1300000839).

#### The FluMum study

FluMum is a national cohort study of influenza vaccine uptake during pregnancy and the effectiveness of pre-natal influenza vaccination in preventing laboratory confirmed influenza in infants. The protocol for this study has been published elsewhere [12]. For this current study, we extracted data on women recruited to the FluMum study for the years 2012 and 2013 who identified as Aboriginal and/or Torres Strait Islander at enrolment.

#### Community-based cross-sectional surveys

We conducted surveys of women presenting to the two participating health services. Whether or not it was standard care at these specific health services to routinely offer and/or provide influenza vaccination to pregnant women was not considered in the choice of recruitment sites as women may present to numerous health care providers (eg midwives, obstetricians, general practitioners etc.) at several different locations over the course of their pregnancy. Women were approached for participation by Aboriginal researchers who explained the study using a plain language statement and written informed consent was obtained. Women who agreed to participate in the survey were asked if they would like to participate in the yarning groups described below and re-consented to that component of the research.

Women were eligible for inclusion in the study if they: identified as Aboriginal and/or Torres Strait Islander; were aged 17 years or more; were between 28 weeks gestation and less than 16 weeks post birth; were willing and able to adhere to all protocol requirements; and, had sufficient verbal English to permit questionnaire completion at study entry. There were no specific exclusion criteria.

At the time of recruitment, participants completed a structured questionnaire with the assistance of the Aboriginal researcher. This sought self-reported influenza status, information relating to the barriers/influences of influenza vaccination, self-reported maternal medical/obstetric history and some socio-demographic indicators. The questionnaire did not specifically relate to whether or not the participating clinics offered the vaccine or whether the offer of influenza vaccination during pregnancy was standard care at those clinics Data were entered into a password protected, electronic database housed at the Queensland Children’s Medical Research Institute.

### Sample size and statistics

The cross-sectional survey was undertaken as a convenience sample. No formal sample size calculations were undertaken given this was a pilot study. Our primary outcome and analysis was the proportion of Aboriginal and/or Torres Strait Islander women who self-reported that they received an influenza vaccine during pregnancy. Secondary outcomes included perceptions of influenza and influenza vaccination in pregnancy. Multivariate logistic regression models were planned to identify potential independent predictors of vaccine uptake during pregnancy if sufficient women were recruited to allow meaningful analysis.

#### Yarning circles methods

The qualitative arm of the study recognized the social context of human behaviour and theorized how norms, routines and patterns of practice develop within those contexts. It suggested that an individual’s knowledge or reality is a social product derived from relations with others through temporal and contextual interactions that influence and determine meanings and actions [16]. This approach is directly suited to studying and analyzing how Aboriginal and Torres Strait Islander women experience pregnancy and vaccination, as well as contextual accounts of decision making around vaccination. Narrative inquiry guided the process of this study [17,18]. We aimed to enroll a maximum of eight participants per group (four groups in total). If saturation point had not been reached following these four groups, a further two groups were to be conducted.

### Data collection and participants

‘Yarning circles’ [15] were held in settings and at a time convenient to participants. Semi-structured sessions began with time for the facilitator to build relationships and rapport with the participants to provide a safe space to share and consider views in the context of the views of others [19,20]. Sessions were audiotaped and a research assistant was present to observe and take notes during the session. A semi-structured interview guide, based on the “*Theory of Planned Behaviour”* [21] was used to inform the conversations. This theory is an accepted approach to understanding behavioural choices of Aboriginal people [22]. The following trigger questions were employed:

1. Can you tell me about what it means to you to be pregnant, or for your family member to be pregnant? (*exploratory*)
  Consider partners, stage of life, other siblings?
2. What has your experience been with influenza?
3. How do you feel about vaccinations? (*attitudes*)
4. How do you see vaccinations during pregnancy? (*attitudes*)
  How do you think others in your community, such as partners, friends, Aunties, view vaccination during pregnancy? (*subjective norms*)
5. Can you think about any things that would influence you to have or not have vaccinations during pregnancy? (*perceived behavioural control*)
6. In relation to being pregnant, can you tell me about services that you attend or don’t attend? *(subjective norms and perceived behavioural control*)
  Why do or don’t you attend these health services? (*perceived behavioural control*)
  What does the ideal health service that you might go to for vaccination look like and what is important about them?

### Data analysis

All yarning circles were transcribed verbatim. The original per protocol analyses of the transcripts included detailed deductive and inductive processes and plans to identify and define underlying patterns across the stories with an emphasis on social, cultural and historical context. However, given the unanticipated small number of participants recruited, narrative summaries of the transcripts and major themes are provided here.

## Results

### Quantitative data

A total of 741 women were recruited to the FluMum study in Brisbane in 2012 and 2013. Of these, 16 (2%) identified as Aboriginal and/or Torres Strait Islander. Sixty-five women were screened in the community-based survey and 37 women were enrolled between January and April 2014. Reasons for non-participation in the community study included time constraints, the partner not wanting involvement, ineligibility and inability to contact after initial screening. This provided data on a total of 53 women for inclusion in the analysis. The demographic and pregnancy status of these women are presented in Table 1.

**Table 1:** **Characteristics of study participants (N = 53), Influenza immunisation project, Caboolture and Toowoomba, 2014**

	***Total***
Median Age in years (Range)	25 (17 – 39)
English as primary language	53 (100%)
Employment status during pregnancy	
Full time	9 (17.0%)
Part time	3 (5.7%)
Casual	5 (9.4%)
Not in paid employment	36 (68.0%)
Education status	
Post-school qualifications	13 (24.5%)
Completed secondary school	19 (35.8%)
Did not complete secondary school	18 (34.0%)
Did not respond	4 (7.5%)
Pregnant at enrolment	19 (36%)
Gestational age (weeks) at enrolment	35 (28 – 40)
Gestational age at first antenatal care visit	9 (5 – 32)
Gestational age at birth for infant	38 (35 – 40)
Ever received influenza vaccine	18 (34%)

### Knowledge of influenza

All but two women had heard about influenza and 18 (34%) women reported they had received an influenza vaccine at some time in the past. Twenty-one (40%) women reported someone had spoken to them about influenza during their pregnancy and some of these women reported several sources. Fifteen of these women reported they had received that information from their general practitioner (GP), 14 from a midwife and seven from other sources (nurse, partner, not specified, obstetrician). Thirty-nine (39/53) women reported that they thought getting influenza during their pregnancy was a serious disease.

### Influenza vaccination during pregnancy

Although nine (9/53) women reported receipt of an influenza vaccine during their pregnancy, 26/53 said they knew influenza vaccine was recommended during pregnancy. Most women (28/53, 53%) said they would accept the vaccine if it was offered during their pregnancy, seven indicated they would not, five did not know and the data were missing for the remaining seven women.

Whereas nine women said they had received an influenza vaccine in their pregnancy, 23/53 reported they were offered the vaccine. Only five of the women who were offered a vaccine actually received a vaccine during their pregnancy (an uptake rate of 22% amongst those that were offered the vaccine). There were 25 women who reported that they were not offered the vaccine during their pregnancy and four women who did not answer the question. Only three of the 25 women who were not offered the vaccine in their pregnancy said they would not have accepted the offer. Two women reported someone had recommended they not get the influenza vaccine during their pregnancy, one by a midwife and one by a grandmother.

In contrast to the 17% uptake rate for influenza vaccination during the most recent pregnancy, 25/53 women reported they would get the influenza vaccine if they became pregnant again, nine said they would not, six did not know and the data were missing for 13 women.

### Perceptions of influenza vaccine in pregnancy

Participants were asked a series of questions about their perceptions of influenza vaccine in pregnancy and immunisations in general (Table 2). Few women, regardless of their pregnancy vaccination status, thought the vaccine would stop them from getting influenza in their pregnancy or that it would stop their newborn from getting influenza. From a safety perspective, only two of nine vaccinated women thought a vaccine during pregnancy could make them sick compared with 22/42 unvaccinated women. Very few women in either group thought vaccination in pregnancy could harm the unborn baby (Table 2).

**Table 2:** Perceptions of influenza and influenza vaccination*, Influenza immunisation project, Caboolture and Toowoomba, 2014

**Do you think:**		**Vaccinated in pregnancy**	**Unvaccinated in pregnancy**
		**n = 9 (%)**	**n = 42 (%)**
Vaccine will stop you getting flu in pregnancy	Yes	3 (33%)	15 (36%)
	No/Unk	6 (67%)	27 (64%)
Vaccine in pregnancy will lessen severity of flu	Yes	4 (44%)	30 (71%)
	No/Unk	5 (56%)	12 (29%)
Vaccine in pregnancy could make you sick	Yes	2 (22%)	22 (52%)
	No/Unk	7 (78%)	20 (48%)
Vaccine in pregnancy is worse than flu in pregnancy	Yes	0 (0%)	5 (12%)
	No/Unk	9 (100%)	37 (88%)
Vaccine in pregnancy will stop newborn getting flu	Yes	2 (22%)	11 (26%)
	No/Unk	7 (78%)	31 (74%)
Vaccine in pregnancy could harm unborn baby	Yes	1 (11%)	6 (14%)
	No/Unk	8 (89%)	36 (86%)
***Which of the following statements apply to you?***			
Think all vaccines are safe		6 (66%)	17 (40%)
Worried about vaccines in pregnancy		3 (33%)	9 (21%)
Allergic to flu vaccine		0 (0%)	0 (0%)
Objects to vaccination in pregnancy		0 (0%)	1 (2%)
Plans to immunise baby		7 (78%)	41 (98%)
Rarely gets sick		3 (33%)	18 (43%)
Doesn’t think flu is serious		1 (11%)	0 (0%)
Can’t afford the cost of flu vaccine		0 (0%)	1 (2%)

*excludes data on 3 women for whom vaccination status was unknown.

#### Yarning circles results

Three yarning circles were held between January and April 2014. While at least 10 women were consented for each group, and planned to attend, only seven in total participated across all three groups. The majority of reasons for non-attendance were related to pregnancy factors (birth, complications, tiredness) or changes in personal circumstances. Participants ranged in age from 21 to 34 years and all were mothers of two or more children. One participant was a practising Aboriginal Health Worker.

### Feelings about pregnancy

One participant expressed she “loved being pregnant” however others voiced feelings of isolation, heightened levels of stress, lack of support, concerns about the impact of their pregnancy on other family members and that they were struggling emotionally. Regular in-home support for women experiencing difficulties during their pregnancies was seen as a particular need that was not being met.

*“I just need basically someone like to sit down to talk to every now and then and but I did have friends, they just changed in what they do…..”*

### Experience with influenza and influenza vaccination

All participants were aware of influenza and had either been ill themselves or reported illness amongst their family members. Overall the participants were supportive of influenza vaccination during pregnancy, and in general, particularly if it was thought to benefit both the mother and the unborn child.

*F33 “Yeah, I basically find it’s very important to have it that is like, it’s not mainly for your health, but if you’re like your kids end up gettin sick too, it’s good for them to have it too… ….like when you don’t have it you’re more sicker than when you do have it…like it calms it down a lot…..”*

Participants also thought that influenza vaccination was important for family members and most thought that even if it didn’t prevent influenza, the vaccine may lessen the severity of disease. They were interested in the safety of the vaccine, what products were used to make the vaccine and wanted to understand the risks of vaccination to self and the foetus, this information is being provided as part of study feedback. All women indicated they would recommend influenza vaccine during pregnancy to friends and family, and all but one indicated they would be willing to be vaccinated. The participant who said she would not be immunized ascribed this to complications in a past pregnancy. However, she was supportive of others getting the vaccine, a comment that was not explored further in the group discussion.

Five of the seven participants were not aware that influenza vaccination was recommended and available free for pregnant women. The majority reported their health service providers had not discussed influenza vaccination with them during their pregnancies. There was a need for more health education, awareness and promotion around influenza vaccinations for pregnant mothers dedicated specifically to Aboriginal and Torres Strait Islander peoples and there was not enough information in either the community or hospital setting. Two members discussed doctor-led education sessions, which would strengthen the relationship between doctors and patients, with patients obtaining information from the source. Other areas discussed included awareness of the ingredients of the influenza vaccine and its benefits. One participant indicated that on top of just providing information, services should just give the vaccine at the time the person was there as the steps required to get vaccinated (ie go to pharmacy, come back to clinic etc.) were difficult to complete given competing priorities.

*F21 “Ummm now when you go in there they have to give you all these descriptions and all that but they don’t do nothing about it… they should just say if you wanted to get the needle, they should just pull out the needle …”.*

When thinking about what would influence them to be vaccinated during pregnancy, the majority considered the potential benefit to themselves and their unborn child as the primary factor.

### Health services

The “ideal” setting for participants with respect to pregnancy care and getting vaccinated during pregnancy was one that was culturally specific, multi-disciplinary, provided external support services and, importantly, one in which there was more active involvement of doctors in discussing vaccination with them. The relationship with doctors was a recurring theme, with participants often discussing the limited interaction and communication they had with their doctors and how they wanted to hear more from the doctor, not others, about vaccination during pregnancy.

*F32 “…somewhere, where you get treated like a person and not just a number I suppose, rather than a than being a cattle prod, so to speak. So get you in get you out… be done with it…. Someone that’s gonna actually gonna look at it as a holistic not only just look at you for being there for a flu vax, ok, your pregnant lets tap into these services………… ok you’ve got umm diabetes, let’s do this, let’s do a health assessment, I want the full coverage for the best for myself, for my family for my baby…so I want a service that’s actually going to provide a holistic point of care…….”.*

## Discussion

This pilot study examined the uptake of influenza vaccine during pregnancy amongst Aboriginal and Torres Strait Islander women from two urban/inner regional communities in South East Queensland. We found that less than half of these women were offered influenza vaccine whilst they were pregnant and only a small proportion had actually been vaccinated. Forty-seven percent of this small sample of women indicated they would get vaccinated if they became pregnant again but this would still leave half of those women unvaccinated. Very few women reported that they thought influenza vaccination in pregnancy would prevent influenza during their pregnancy nor in their newborns. While our sample size was small our study confirms a need for larger studies.

Our findings with respect to low uptake of influenza vaccination in pregnancy are similar to studies conducted in other populations, albeit with coverage ranging from 10 to 60% [23-27]. Studies that have investigated differences in uptake within populations have reported lower uptake in minority groups (eg non-Hispanic African Americans compared to non-Hispanic Americans in the United States) and between socio-economic groups [24,28-31]. Potential explanations for these disparities include access to health services, cost of vaccine and the logistics of being vaccinated and the lack of socio-culturally appropriate education. The qualitative data arising from the yarning circles seems to support the latter two factors as important determinants of vaccine uptake. A lack of adequate information about the vaccine, why it is needed and its safety in pregnancy have been identified as major factors behind pregnant women declining a vaccine offer [24,31,32]. The majority of both vaccinated and unvaccinated women in our study did not believe the vaccine would prevent influenza in pregnancy or that it would prevent influenza in their newborns. This suggests the information that is available to women may not adequately address these issues and that they may not be discussed in detail with providers at the time vaccine is recommended or offered.

How women feel about pregnancy may also be a factor in the conversations about health including immunisation. As one woman identified: *“I just need basically someone like sit down to talk to every now and then”….* Further exploration is required about how the decision about influenza immunisation interplays with other life issues for women who are pregnant.

The importance of the recommendation for vaccination by a woman’s practitioner, particularly the medical practitioner, has been documented in several studies [24,26,31,33]. The level of knowledge a physician has about influenza vaccination during pregnancy has also been reported to be associated with vaccine uptake [34]. Our focus group participants indicated the importance of the medical practitioner in discussing vaccination during pregnancy. Participants suggested they preferred to receive the information from their doctors than others and that doctors needed to be more involved in discussing vaccination with their patients.

Less than a quarter of the women in our study who were offered the vaccine (and would have accepted it) were actually vaccinated. While the survey did not ask why they were not vaccinated despite an offer of vaccine, our focus groups suggest that the vaccine not being immediately available at the time of the offer may play a role. The need to take a script to a pharmacy, collect the vaccine and return to a clinic for a second time to be vaccinated was a stated deterrent. Provision of vaccine at the time the recommendation or offer is made is likely to facilitate vaccine uptake, particularly in outpatient settings where accredited nurse immunisers are available to administer the vaccine [35].

The predominant strength of this study was the involvement of Aboriginal research staff within the two participating health services and the associated follow-up. This provided a culturally appropriate approach to data collection, capacity building for Aboriginal and Torres Strait Islander staff, students and health service providers, and identified factors that would need to be considered in future studies. The combination of both quantitative and qualitative data provided a richness of data that could not be achieved by one method alone.

The major limitation of this study is the small sample size. While larger numbers were planned for both the community survey and the yarning circles, this was not achieved within the available time frame. The predominant barriers to recruitment and to completion of the yarning circles were either ineligibility or competing priorities for potential participants. However the study was by design an exploratory pilot study that has identified key issues needing further investigation in larger studies.

A further weakness of this study is that we relied on self-report of vaccination status. There are limited studies that have evaluated the validity of self-reported antenatal immunisation amongst pregnant women [36]. The limited data available on the reliability of self-report, and none for Australian Aboriginal and Torres Strait Islander women, is a limitation to interpreting coverage data and monitoring the effectiveness of immunisation campaigns, particularly when population-based adult immunisation registers are unavailable. Further studies need to incorporate validation of self-report into study procedures.

## Conclusion

While the findings of our small study cannot be considered representative of Australia’s Aboriginal and Torres Strait Islander population, it suggests influenza vaccination uptake during pregnancy in Aboriginal and/or Torres Strait Islander women may be low. There is a lack of knowledge of the recommendations for vaccination, health service providers are not universally offering the vaccine and importantly, women are not being vaccinated even if the vaccine was offered. Women reported wanting to hear more from doctors in regard to influenza immunisation.

While our yarning circles suggest there is a lack of information available to women, and that the logistics of being vaccinated even if it is offered or they know it is recommended, and these are likely to play an important role in vaccine uptake, our findings point to an urgent need to repeat the study on a larger scale and in a broader cross-section of communities. In addition to exploring in more detail the reasons why women are not vaccinated even if offered the vaccine, an important question to address is the acceptance of influenza vaccination during pregnancy in Aboriginal and Torres Strait Islander women given approximately half of our study population indicated they would not be vaccinated in their next pregnancy.
